# The role of antioxidants in improving biodiesel's oxidative stability, poor cold flow properties, and the effects of the duo on engine performance: A review

**DOI:** 10.1016/j.heliyon.2022.e09846

**Published:** 2022-07-03

**Authors:** Nurul Aini Amran, Usman Bello, Muhammad Syafiq Hazwan Ruslan

**Affiliations:** a Chemical Engineering Department, Universiti Teknologi PETRONAS, Seri Iskandar 32610, Malaysia; b HICoE—Centre for Biofuel and Biochemical Research (CBBR), Universiti Teknologi PETRONAS, Seri Iskandar 32610, Malaysia; c School of Chemical Engineering, College of Engineering, Universiti Teknologi MARA, Shah Alam 40450, Malaysia

**Keywords:** Biodiesel, Oxidative stability, Cold flow, Antioxidants, Engine performance

## Abstract

Global competitiveness thrives on meeting energy demand, and the need to counter the effects of environmental threads dispatched by the combustion of fossil fuels became the driving forces that upended the renewed commitment and growing interest in renewables. Alternatively, green energy provides a twofold solution to energy and environmental crisis in a sustainable, economically viable, and eco-friendly manner. However, energy from biomass, especially biodiesel is considered an attractive substitute for mineral diesel, with the proficiency of meeting future energy demand. Inevitably, biodiesel exhibits poor cold flow properties leading to plugging and gumming of filters, whereas oxidation stability results in sediments and gum formation. These effects present a legitimate concern to producers and the automotive sector. Many reviews on the use of antioxidants to improve biodiesel's cold flow and oxidative stability flooded the literature independently. Yet, a review encompassing the factors inducing biodiesel's poor cold flow, oxidation stability, their effects on engine performance, and the inhibitory role of antioxidants appears vacant. Hence, this paper put together the above-stated aspects, with the first part discussing the factors initializing and accelerating oxidation, the mechanism of oxidation, and biodiesel cold flow were subsequently discussed. Next, the inhibitory functions of antioxidants on biodiesel's oxidation stability and poor cold flow were also explained. Finally, this review reflects on the research trends and sustainability prospects of using antioxidants for improving biodiesel's poor flow and oxidative stability without hindrance to the engine system.

## Highlights

• This review discusses the inhibitory role of antioxidants on biodiesel's oxidation stability, poor cold flow, and their effects on engine operation.
• SFAs and USFAs are two concurrent drivers that susceptibly set up biodiesel to undergo oxidation and poor cold flow.
• Poor cold flow and oxidative stability of biodiesel are substantially improved with the addition of antioxidants.
• Antioxidants that are potent at low concentrations, utterly soluble, and non-toxic are more effective in improving biodiesel quality.

## Graphical abstract

**Figure:**
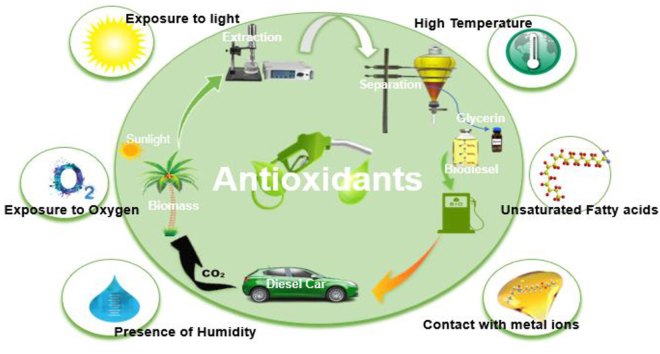

## 1 Introduction

The issues of fossil fuel exhaustion raise much concern considering the proportional increase in energy consumption rate due to emerging economies pooled by rapid population growth, industrialization, and transportation demands. This accelerates the global energy demand and aggravates nature-related hazards linked to excessive fossil fuel utilization. Accordingly, these ever-growing energy demands, coupled with the spike in the spontaneous discharge of greenhouse gas (GHG) emissions emanating from burning fossil fuels, constitute some of the global challenges faced today [1]. This compelled the urgent need for clean, low-cost, and sustainable energy across the globe, giving rise to the intensified exploration of diverse research-based strategies and revised production routes using optimized techniques and remodeled approaches to push for clean energy production in line with ensuring affordable and sustainable energy for all, as advocated by sustainable development goals (SDGs) [2, 3]. This will support the attainment of various regional energy security targets, clamp down on environmental pollution and neutralize the glaring risks of climate change. However, according to the recent updates released at the COP-26 climate summit held in October 2021 in Glasgow-Scotland, Australia has the highest GHG emissions from coal power plants, a two-fold that of China [4]. Other top emitters include the USA and some European countries, whose CO_2_ emissions contribute to that of the world by 85 % [5]. In the same vein, ASEAN which is considered one of the rapidly growing economic regions in the 20th century [6], is primarily dominated by the use of fossil fuels in its energy mix and this makes it one of the frontline contributors to global warming and GHG emitter, with a projection of 2.3 billion tonnes of emissions by 2040, unless significant decarbonization of accelerated coal phaseout takes place [7]. Consequently, in striving to achieve the carbon neutrality target of 2050, energy diversification strategies must be intensified to ensure energy security scale-up before getting rid of fossil fuel usage, considering its massive dent in the global energy mix. Hence, the current global thrive towards low carbon emission sources signaled that the bulk of future energy must be derived from clean and renewable sources.

**Figure 1:**
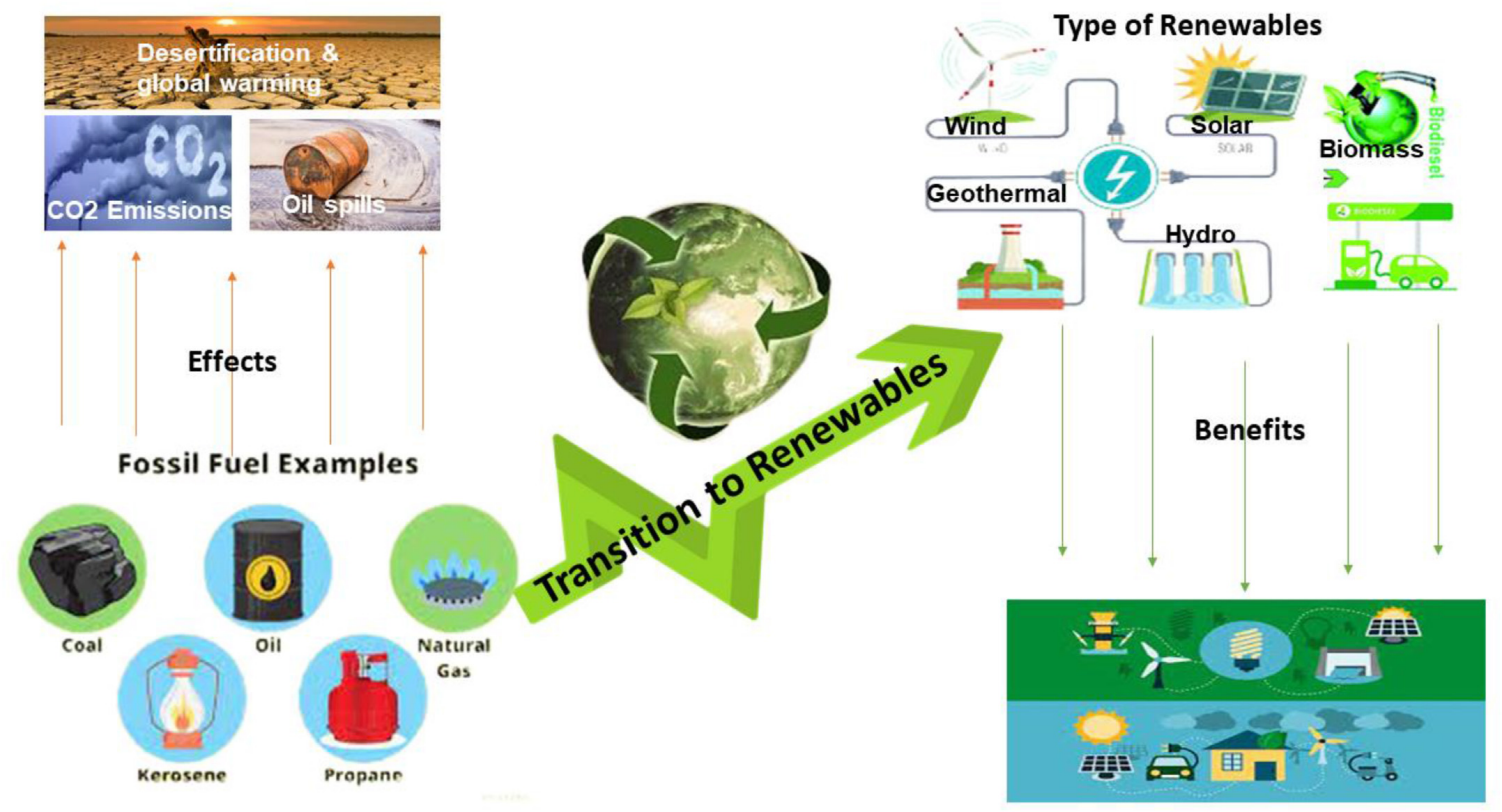
Sustainable energy transition paradigm from fossil fuels to renewables.

Therefore, in the fate of the growing energy poverty, heightened by depletion and the non-sustainability of fossil fuels, transitioning to renewables was born out of the necessity to provide clean energy, fast-track sustainable economic growth, and decelerate the grievous effects of global warming stimulated by GHG emission. Figure 1 shows a sustainable energy paradigm chart from fossil fuel to renewables, and this transition would assist in holding down the extreme weather and climate impacts posed by GHG emissions while at the same time supporting clean and sustainable energy production. Perhaps, biodiesel is considered a plausible and better candidate considering its renewability, recyclability, biodegradability, and sulphur free with a low carbon emission profile [8]. While other renewable energies such as solar, hydro, and the wind are mainly utilized to power the electric grid, energy from biomass, particularly biodiesel, will provide electricity and will also underpin to quench the bulk thirst for the liquid fuel demand by the transport sector.

Sadly, oxidation stability and poor cold flow properties of biodiesel are two concurrent barriers that retarded broader patronage and mass commercialization of biodiesel despite its growing demand, and still, these problems are yet to be adequately dealt with. Although many researchers published review papers on biodiesel oxidative stability [9], techniques to improve biodiesels’ oxidative stability [10], factors affecting oxidation, including some techniques to improve the stability and cold flow [11], and the effects of antioxidants on the stability of biodiesel, performance, and emission of the engine were also reviewed [12]. Surprisingly, despite many available reviews in the literature, a combined discussion on the impacts of antioxidants on oxidative stability, poor cold flow properties of biodiesel, and their effects on the engine system fell short of reporting. Therefore, the present review put together the stated aspects and sampled a few parameters discussed in the present review, which outnumbered those discussed in the existing related review articles upon comparing as presented in Table 1.

**Table 1:** Comparison of the present review with previous existing related review articles.

Literatures Number of parameters discussed	[13]	[9]	[14]	[15]	[11]	Present Review
1. Effects of fatty acid composition on biodiesel from different feedstocks.	˟	11	20	15	20	17
2. Use of antioxidants to improve biodiesel oxidative stability.	˟	20	˟	25	˟	50
3. Use of antioxidants to improve biodiesel cold flow	˟	17	26	˟	˟	13
4. Effects of cold flow behaviour on engine performance.	23	16	˟	˟	˟	20
5. Effects of oxidation stability on engine performances.	˟	04	˟	✓	˟	✓
6. Mechanism of oxidation stability and cold flow properties in biodiesel.	˟	˟	✓	˟	˟	✓

˟ = Not discussed. ✓; = Discussed.

### 1.1 Biodiesel oxidative stability

**Figure 2:**
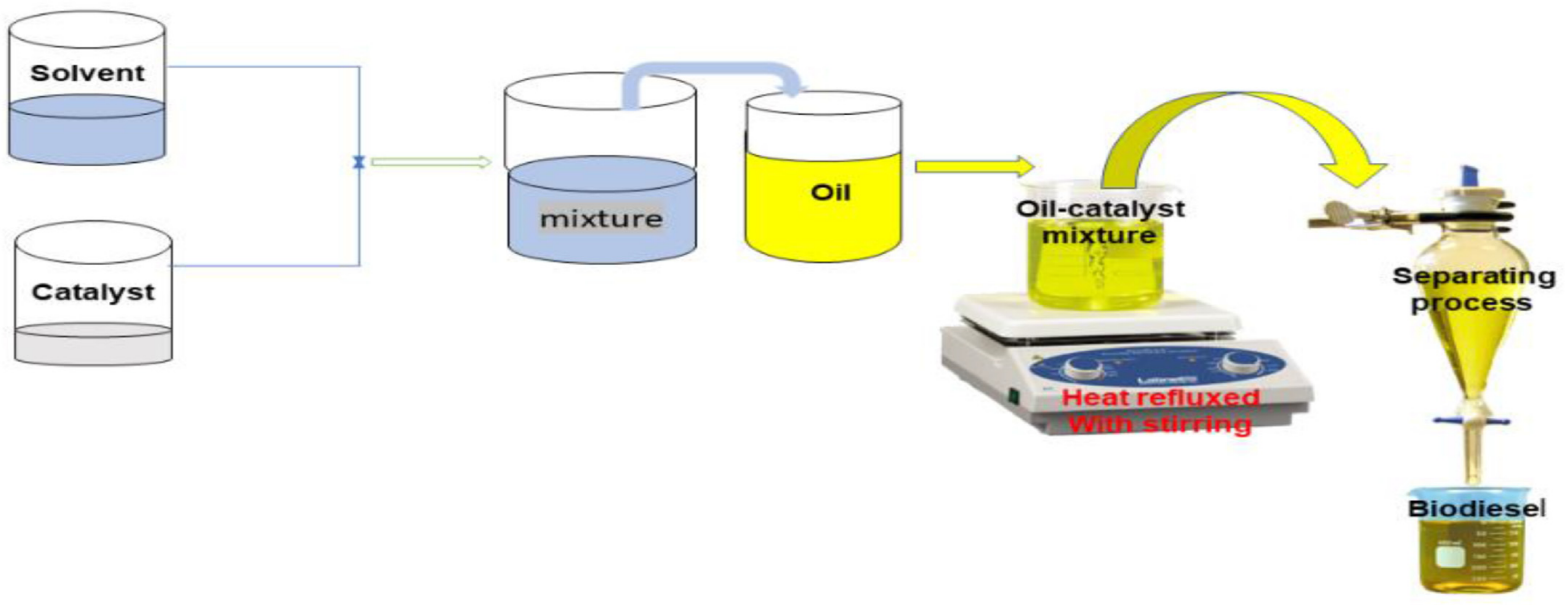
A simple base-catalyzed transesterification process of biodiesel production.

Biodiesel is clean energy that can be produced from edible, non-edible crops or animal fats through the transesterification process. It is produced when oils or fats react with solvents in the presence of catalysts (acid, base, acid-base, or enzymes); to give fatty acids esters (biodiesel) and glycerol, as illustrated in Figure 2. The produced biodiesel is considered a potential alternative for conventional diesel that can be used without further modifications and used directly due to its low toxicity less potency GHG and low carbon emission profile [16].

**Figure 3:**
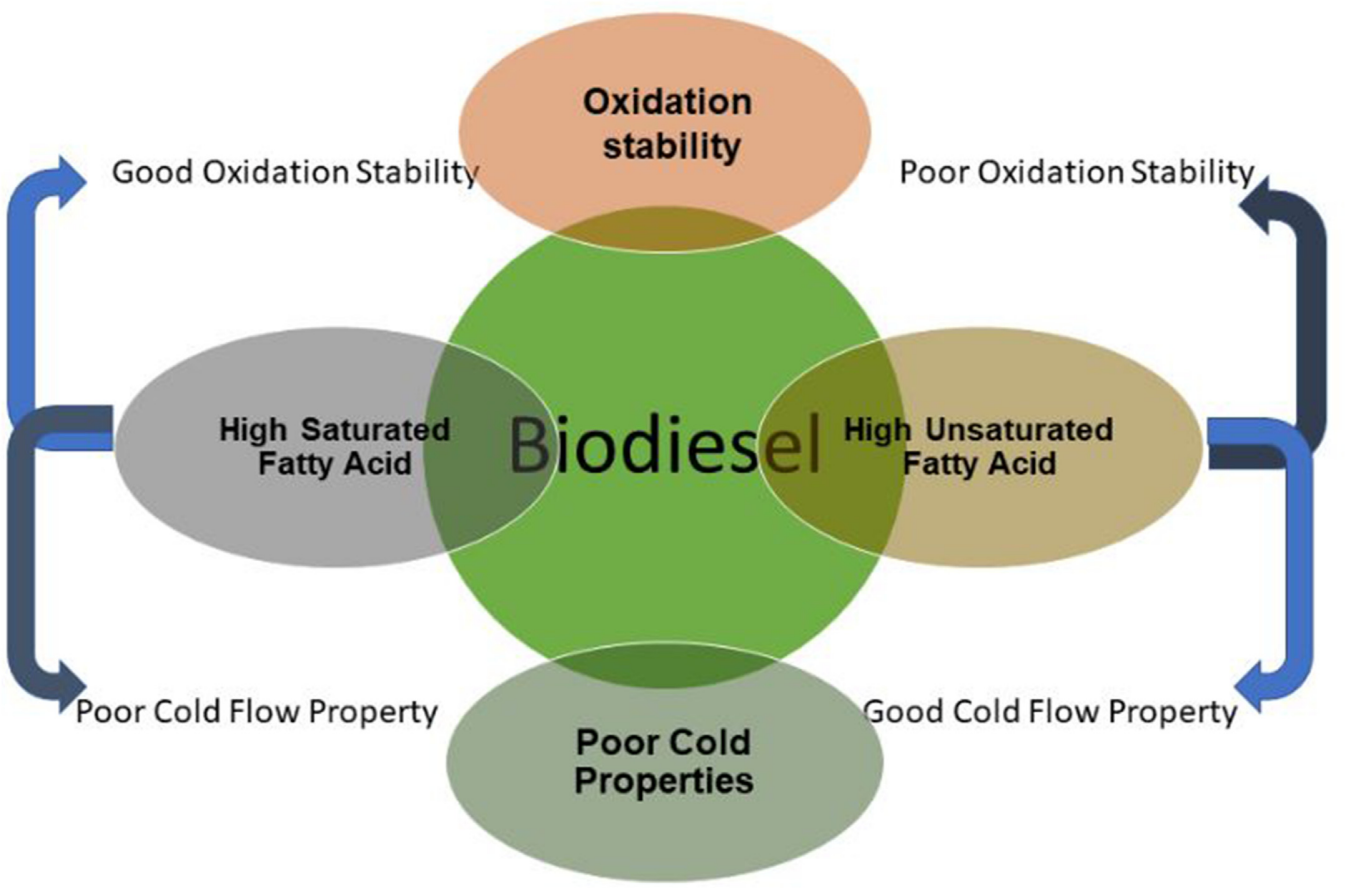
Effects of fatty acids on oxidation and poor cold flow of biodiesel.

However, consumers, particularly those in the automotive industry, lamented that biodiesel's oxidation stability and poor cold flow properties are the two major causes of the decline in fuel quality [17]. Although the two scenarios are mainly plagued by fatty acid compositions present in the feedstock used in producing the biodiesel, some discrepancies occurred between them. Such that biodiesels made from feedstocks containing much quantity of saturated (SFAs) and few unsaturated fatty acids (UFAs) possess high cetane numbers, high calorific value, and better oxidative stability, which are desirous, signifying good fuel quality [18]. Unfortunately, this may set out poor cold flow properties with increased viscosity, thereby antagonizing the fuel quality properties on the other hand through fuel filter clogging and hindering ignition efficiency in combustion engines [19]. In contrast, biodiesel having a high proportion of UFAs and small SFAs possessed a lower cetane number and poor oxidative stability, thereby augmenting the undesirability of the fuel quality. Figure 3 presents an interplay of SFAs and UFAs about how they influence biodiesel's oxidation and poor cold flow. The influence caused by other oxidation actors such as high temperature, metal contaminants, oxygen, and sunlight exposure can be potentially avoided through proper handling and storage [2, 21]. Therefore, injecting raw biodiesel directly into diesel engines is not encouraged because of the above-stated problems and other performance drawbacks like incomplete combustion and poor atomization, leading to engine fouling [21]. These operability issues typically increase service costs and jeopardize equipment safety. Researchers attempted to address these problems through several research findings with numerous techniques adopted, such as blending the biodiesel with conventional diesel, adding antioxidants, micro-emulsification, and pyrolysis. Undoubtedly, the use of antioxidant additives to enhance biodiesel's oxidative stability and cold flow properties is at the centre stage of investigation among researchers [22].

The susceptibility of biodiesel to oxidative degradation is proportional to the nature of fatty acid composition in the feedstock type from which the biodiesel is produced. The chemical structure of fatty acids in the feedstock is linearly related to oxidative instability, despite fatty acid composition differing among feedstock types [23]. Meira et al. [24], identified fatty acid composition, total glycerine, humidity, and fuel storage conditions such as temperature and exposure to light as some of the factors responsible for autoxidation. This oxidation process is initiated by losing hydrogen atoms belonging to allylic or bis-allylic carbon in the presence of an initiator to form a free radical, which quickly reacts with an oxygen molecule to form peroxyl radicals and subsequently propagates to form hydroperoxides. These unstable products (hydroperoxides) disintegrate upon continued propagation to form aldehydes and short-chain acids as oxidation products. These compounds progressively generate insoluble gums through polymerization, increasing acid value, peroxide value, and viscosity [25]. As a result, a series of changes occur when biodiesel is oxidized, often compromising biodiesel quality and hindering engine performance. Therefore, the structural composition of fatty acid and environmental factors such as luminosity, humidity, high temperatures, metal contaminants, and oxygen in the form of singlet and triplet [26], catalyzed the initiation of the oxidation process by generating free radical from the alkyl chain [27]. Below are some of the main oxidation actors, responsible for oxidation stability in biodiesel.

1 Fatty acid

Fatty acids are saturated, such as stearic, palmitic, and hydroxy stearic acids, and unsaturated like oleic, linoleic, ricinoleic, palmitoleic, linolenic, and eicosanoid acids, respectively [28]. Although, SFAs have a negligible effect than UFAs towards vulnerability to oxidation in biodiesel. The accessibility and cost; determine the type of feedstock to be used in producing biodiesel in any country. This promotes the exploration of homegrown raw materials and fosters local content development, while simultaneously cutting down the cost of production. Also, the fatty acid esters and their composition vary based on the feedstocks' sources, as shown in Table 2. The variations in biodiesel composition have direct consequences on fuel quality, engine performance, and exhaust emissions [11]. The Feedstocks containing a very high quantity of SFAs displayed higher oxidative stability than those made up of too much monounsaturated (MUFA) and polyunsaturated fatty acids (PUFA). This could be attributed to the fact that MUFA and PUFA, are more prone to oxidation attack and hence resist the inducements of the antioxidants inhibition effects [22].

2 Storage temperature

**Table 2:** Effects of fatty acid composition from various feedstocks on biodiesel oxidation stability index (Induction Period) determined at 110 °C.

Feedstocks	Palmitic acid (C16:0)	Stearic acid (C18:0)	Oleic acid (C18:1)	Linoleic acid (C18:2)	Linolenic acid (C18:3)	Arachidic acid (C20:0)	Erucic acid (C22:1)	Lauric acid (C12:0)	Capric acid (C10:0)	SFA (%)	MUFA (%)	PUFA (%)	IP (h)	References
Palm oil	40.3	4.1	43.4	12.2	-	-	6.87	0.2	-	44.6	39.0	11.0	17.0	[32]
Sunflower	32.21	9.27	43.7	21.98	-	-	-	0.22	0.51	10.0	21.0	62.0	1.5	[1]
Jatropha	16.68	7.7	39.1	36	0.2	0.2	-	-	-	23.4	39.2	36.2	4.2	[33]
linseed	6.4	4.5	21.7	13.5	52.7	0.2	-	-	-	11.4	21.8	66.2	2.2	[21]
Soybean	11.0	4.0	23.1	53.2	8.5	-	-	-	-	15.0	23.1	61.9	3.0	[34]
P/kernel	8.4	2.4	15.4	2.4	-	0.1	-	47.8	3.5	82.1	15.4	2.4	14.0	[21]
PFO	10.9	2.8	17.3	68.1	0.3	-	-	-	-	14.2	17.4	68.4	13.6	[35]
WCO	7.34	2.01	4.83	1.34	-	0.61	-	49.59	9.98	69.5	4.8	1.34	3.1	[36]
Peanut	8.3	2.6	51.9	21.8	0.3	1.8	-	0.3	-	13.0	52	25.0	2.0	[37]
Canola	5.0	2.0	64.0	19.8	7.2	0.6	0.3	-	-	8.0	64.5	27.0	6.5	[38]
Coconut	6.09	1.9	4.98	0.82	-	-	-	51	7.9	93.8	5.0	0.82	14.2	[39]
Rapeseed	5.95	2.07	60.34	20.87	8.15	0.61	-	-	-	06	60.3	29.0	2.0	[40]
Karanja	11.6	7.5	51.5	16	2.6	1.7	-	-	-	12	68	13.0	3.9	[41]
MOME	2.9	5.5	74.1	4.1	0.2	2.3	2.8	-	-	18.6	77.1	4.3	-	[42]
APME	18.4	11.8	18.3	26.7	23.2	0.5	-	-	-	30.7	19.4	49.9	0.2	[43]
CIME	12.01	13.0	34.09	38.26	0.3	-	-	-	-	25.0	34.1	38.6	6.1	[22]
SME	10.2	3.7	22.8	53.8	8.6	0.3	-	-	-	14.5	22.8	62.3	4.1	[22]

MOME: moringa oleifera methyl ester, APME: aphanamixis polystachya methyl ester, CIME: *Calophyllum inophyllum* methyl ester, SME: sesame oil methyl ester, PFO: poultry fat oil, WCO: waste cooking oil.

Many studies identified temperature as a key promoter of biodiesel oxidation stability [18], such that an increase in temperature, raises the possibility of oxidization in biodiesel. However, the correlation between increased temperature and oxidation stability is linear and un-deviated from the degradation rate. Thus, low-temperature storage condition minimizes the rate of biodiesel degradation [29]. Typically, the breakdown of the fatty acid chain and the formation of a volatile product during oxidation is accelerated upon exposure to sunlight and higher temperature.

3 Metals contaminants

The presence of metal often arises due to activated enzymes or their decomposition products. These metal ions get into the biodiesel during production, refining, or other processes like hydrogenation and accelerate the rate of oxidation even at small concentrations. Therefore, pro-oxidant metals such as Co, Cu, Fe, Mn, possessing two or more oxidation states, bear a suitable oxidation-reduction potential and quickly reduce the length of the induction period (IP) [30].

4 Exposure to light

The oxidation rate is usually sped up when biodiesel is subjected to light by infiltrating and expanding the segment of fatty esters, thereby deteriorating the quality of the fuel. However, the effects of light on the oxidation stability of biodiesel depend on fatty esters compositions, type of antioxidants additive, and other sensitizers [31].

#### 1.1.1 Mechanism of biodiesel oxidation stability

The mechanism of oxidation proceeds via autooxidation, photooxidation, thermal, and enzyme induced. However, autoxidation is identified as the commonest form through which oxidation occurs, and is mainly brought about due to the predominance of UFAs present in the feedstock [44]. Therefore, the mechanism for forming oxidation stability in this paper would dwell on autoxidation since the other pathways impart lesser effects.

#### 1.1.2 Autoxidation

**Figure 4:**
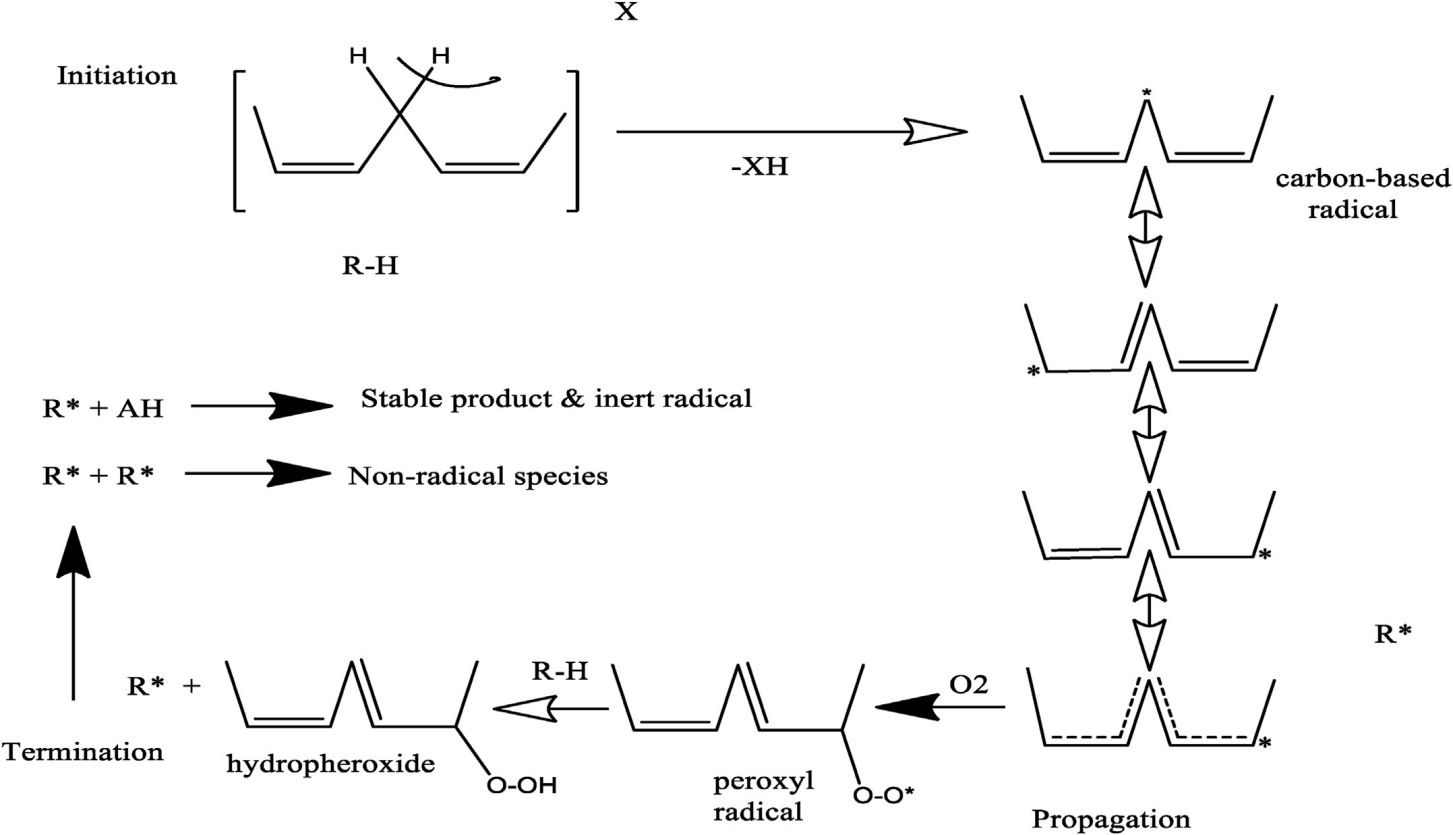
Three phases of the autoxidation process.

This is a self-catalytic reaction of molecular oxygen which can be sped up by exposure of biodiesel to high temperature, air, or light, leading to the formation of polymeric products that affects the biodiesel quality. Therefore, the composition of the fatty acids, structural form, processing, and storage conditions, dictate the extent of the auto-oxidation reactions [38]. Typically, free radicals are some of the intermediates formed in autooxidation, and these are sets of atoms that bears an odd number of electron or possess one or more unpaired valence electrons that exist freely for a short time. Hence, the free radical autooxidation mechanism is generally considered the quickest pathway for rancidification, and this involves a series of reactions proceeding through initiation, propagation, and termination phases, as presented in Figure 4.

##### 1.1.2.1 Initiation stage

When biodiesel encounters initiators during processing or storage, a hydrogen atom belonging to either allylic or bis-allylic (carbon flanked by two allylic carbons) carbon is lost and formed a carbon atom with an odd number of electrons (free radical) that is exceedingly reactive (Eq. 1).

Eq. 1 RH + Initiator → R^•^

The carbon radical is a product of decomposition from the lipid and is produced due to contact with the initiators such as high temperature, light, humidity, atmospheric air, or metal ions. Unfortunately, the actual mechanism that produced the initiator free radicals is still unaccounted for. However, the direct reaction of lipid with molecular oxygen (O_2_), usually disobeys the principle of conservation of spin angular momentum. This is because lipid molecules are in a single electronic state while the molecular oxygen is uncertain in its ground state. Therefore, the molecular oxygen and lipid reaction to form hydroperoxides (Eq. 2), is often not feasible due to the high activation energy 146–272 kJ/mol [45].

Eq. 2 RH + ^3^O_2_ → ROOH

Also, Arbos et al. [46], revealed that photosensitizers absorb light in the UV region, such that their molecules progressed from lower to higher energy levels, and transfer their excess energy to an oxygen molecule. Typically, when sensitizers get excited, triplet oxygen is reduced to singlet oxygen, which further reacts with lipid to form hydroperoxide as shown in Eq. (3).

Eq. 3 RH + ^1^O_2_ → ROOH

In similar research, Ghoreishi & Moein [47], estimated the activation energy of the oxygen-oxygen bond in ROOH to be 184 Kjmol^−1^ and concluded that the bond is relatively weak and can therefore be dissociated to form two different radicals (alkoxyl radical and hydroxyl radical) as shown in Eq. (4).

Eq. 4 ROOH → RO^•^ + ^•^OH

Moreover, reactions with metal ions also catalyzed the decomposition of hydroperoxides in lower and higher oxidation states.

Eq. 5 ROOH + M^n+^ → RO^•^+ OH^•^ + M^n+1^

Eq. 6 ROOH + M^n+1^ → ROO^•^ + H^•^ + M^n+^

Therefore, Eqs. (5) and (6), are very sensitive to the generation of free radicals, especially when a trace quantity of metal is added, however, Eq. (6) may be insignificant towards autoxidation.

##### 1.1.2.2 Propagation stages

The obtained lipid radical is very reactive and readily undergoes regeneration reactions by abstracting another hydrogen atom as in Eq. (7).

Eq. 7 RH + R^•^ → R^•^ + R^•^ H

Or by reacting with molecular oxygen (O_2_) in its ground state to generate peroxyl radical as shown in Eq. (8).

Eq. 8 R^•^ + O_2_ → ROO^•^

The reaction of the oxygen molecules with the lipid radical is a swift reaction with zero activation energy (Eq. 8). The produced peroxyl radical (ROO^•^) is less reactive than the carbon-based free radical (R^•^), but further reacts and propagates to the second step by quickly abstracting another hydrogen from a methylene moiety to form hydroperoxide (ROOH) and another carbon radical as shown in Eq. (9), which is subject to fragmentation easily by heat or UV-light [26].

Eq. 9 ROO^•^ + RH → ROOH + R^•^

The propagation continues when the new alkyl radical (R^•^) reacts with molecular oxygen and keeps propagating endlessly. This second step proceeds slowly until the termination, producing stable products [21]. However, the hydroperoxides are unbalanced and therefore can disintegrate to produce secondary oxidation products such as alcohols, aldehydes, and shorter-chain carboxylic acids, which can undergo reactions such as aldol-condensation and polymerization to form insoluble, gums and sediments. Upon the formation of the aforementioned substances, some of the biodiesel quality parameters like viscosity and acid number are severely affected.

##### 1.1.2.3 Termination

At termination, the reaction can be ceased by eliminating the earlier produced radicals, through a recombination reaction between the radicals to form monomers that transform them into non-radical and stable products as shown in Eqs. (10) and (11). Substances such as aldehydes, ketones, alcohols, ethers, organic acids, and oligomers are liberated from the reactions.

Eq. 10 R^•^ + R^•^ → R-R Non radical species

Eq. 11 ROO^•^ + ROO^•^ → Stable products

### 1.2 Effect of biodiesel oxidation stability on engine performance

Biodiesel is considered not trouble-free alternative fuel due to its oxidation stability, characterized by increased deposits on injectors, compromised pressure drops across filters, and hindered free flow. Thus, prompting many researchers to investigate the problem of oxidation in engine operation [48]. However, a high amount of UFAs susceptibly brings about biodiesel oxidation stability, liberating unwanted side products such as acids, aldehydes, ketones, etc, which subsequently form insoluble sediments, and gums. These compounds usually provoked the viscosity, acid number, and cetane number of the biodiesel, which later downgraded the fuel's quality by displaying abnormal flow, corroding fuel lines, plug fuel filters, and clog injectors properties, and ultimately inhibiting smooth engine performance. Warner & Moser [49], reported that a high amount of UFAs in WCO, rapeseed, palm, and soybean biodiesel subjected the fuel to oxidation. The use of biodiesel containing a higher cetane number in a multi-cylinder diesel engine operating at a constant speed, lead typically to reduced carbon monoxide (CO) and hydrocarbon (HC) emissions. On another part, feedstocks like soybean, jojoba, chicken fat, and grease oil have the least NOx emission profile while sunflower, Karanja, fish oil, and WCO have low smoke emissions [50]. Studies carried out by Jakeria et al. [52], reported that due to the hydrophobic nature of biodiesel, contact with water degrades elastomers by corroding metallic components of the engine.

Consequently, this deteriorates the quality of the fuel by raising the total acid number, thereby rusting fuel lines and nozzles upon exposure to air [53]. Moreover, prolonged oxidization reaction of biodiesel leads to polymerization reaction, giving rise to increased molecular weight and viscosity. As a result, the development of insoluble occurs during storage which ultimately obstructs fuel lines, blocks fuel pumps, and increases engine wear. Ochi et al. [55], examined the performance of coconut, palm, jatropha, and soybean biodiesels on the diesel engine. According to the authors, coconut biodiesel showed lowered ignition delay owing high amount of SFAs compared to other biodiesels. An investigation of biodiesel made from edible and non-edible oils, mainly coconut, palm oil, rapeseed, soybean, sunflower oils, cottonseed, jatropha, jojoba, and Karanja oils respectively, was reported by Rajak & Verma, [50]. According to their investigations, a significant difference in the emission characteristics among the biodiesel types was due to the variation in fatty acid composition, resulting in different fuel properties.

### 1.3 Biodiesel cold flow properties

Poor cold flow properties such as cloud point (CP), pour point (PP), and cold filter plugging point (CFPP) severely affects biodiesel quality parameters. These properties are significantly less satisfactory than those in mineral diesel [13]. Typically, the crystallization of biodiesels affects the engine's operability in cold climatic conditions. This could be attributed to the high viscosity, high density, poor atomization, and vaporization, which compromised the engine fuel system by choking the fuel filters and blocking fuel inlet lines and nozzles [56]. Therefore, monitoring the variation of the fuel quality parameters is crucial, especially before the fuel is exposed to specific environmental conditions. Even though ignored by many biodiesel producers, the cold properties are requisite to the longevity and better performance of the fuel, for avoiding CFPP and low-temperature filterability [57]. Thus, neglecting these properties, especially in a low-temperature setting, may lead to the formation of crystals by the fuel. Hence, viscosity, pouring ability, filtration capacity, and fuel volatility are at stake. This gave rise to fuel starvation which subsequently affect the ease of engine starting [14]. According to Islam et al. [58], low-temperature flow fosters serious consequences to biodiesel, especially the one containing a higher amount of SFAs. In such instances, biodiesel faces the threat of being crystallized during winter, resulting in fuel flow restriction and operability problems as crystalline substances block fuel lines and filters. Therefore, many researchers established that a higher number of saturated components of the fatty acids, provoked CP, and PP, due to their high melting point at low temperatures. Typically, crystals appeared at certain temperatures below average in the fuel, thereby inhibiting the free flow of the biodiesel.

At low ambient temperature conditions, cold flow is a property of concern in biodiesel, and this made researchers intensify efforts towards enhancing biodiesel flow properties at low temperatures, particularly by assessing CP and PP. This is to unlock the potential of biodiesel and further broaden its scope of acceptability and its large-scale commercialization, especially in winter-prone environments. Therefore, the lowest temperature at which a liquid specimen becomes cloudy or the temperature at which crystals appear in the fuel is called CP. Hence, below that of crystal formation, a network of crystals is developed from the fluid that prevented it from flowing at specific temperatures. Thus, the lowest temperature at which fuel ceases to flow or becomes pourable, thereby losing its flow characteristics, is known as the PP [13]. The PP of biodiesel can be measured according to the ASTM standards D5949, D5950, D5985, D5985, D6749, D6892, and D97. Consequently, CP and PP are the two low-temperature properties of fuels, which are the criterion for assessing the suitability of fuel for use in engines under certain environmental conditions, such that the temperature of PP is always lower than the CP.

#### 1.3.1 Mechanism of biodiesel cold flow properties

**Figure 5:**
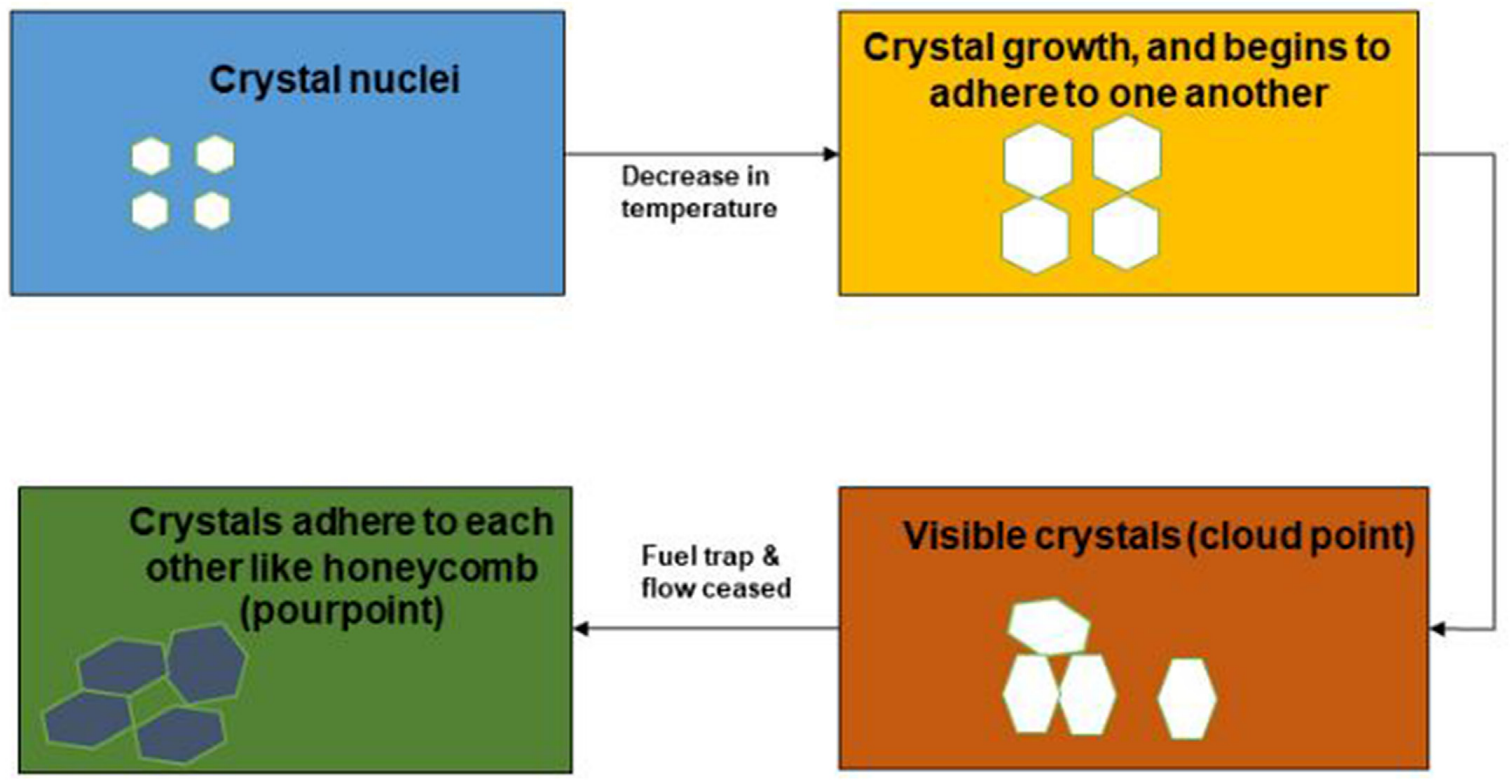
Mechanisms of the cold flow in biodiesel.

Crystal growth and nucleation are stages related to the formation of crystals in biodiesel [59]. In nucleation, when the melting point exceeded the temperature of biodiesel, liquid molecules produced adequate thermodynamic force by strong van der Waals force of interaction, hence crystallization occurred. Typically, poor cold leading to crystal formation in biodiesel is mainly brought about due to the high quantity of SFAs, by having a high melting point at very low temperatures [53]. When biodiesel made from such feedstock is exposed to low ambient temperature, tiny crystals are formed, becoming more pronounced upon further decreases in temperature [60]. These crystals adhere to one another and metamorphose to form visible crystals (CP) with lattice that traps the oil. At this stage, crystal growth after nucleation propagates upon forming an interlinked network by adhering to one another, leading to fuel starvation and flow restriction (Figure 5). When the biodiesel ceased to flow, the PP occurred. This solidification in the fuel occurs quickly, leading to bunch formation that clogs fuel lines and filters, and affects engine performances [61]. Therefore, biodiesel made from higher UFAs feedstocks possesses a lower melting point than saturated compounds. Whereas, biodiesel made from high SFA compounds is prone to displaying higher cloud and pour points.

### 1.4 Effect of biodiesel cold flow on engine performance

The problem of engine operation during cold weather has been of concern, with researchers working towards finding a solution to such issues. Problems affecting the engine related to cold flow properties are mainly plugging of fuel filters and fuel lines leading to flow restrictions; difficulty in starting, clogging of fuel filters, causing poor atomization and vaporization; inadequate burning, and incomplete combustion [20]. These effects of cold flow properties of biodiesel are analogous to the high amount of SFAs, and the length of the hydrocarbon chains present in the feedstock. A detailed summary of the crystallization behaviour of different biodiesel samples is presented in Table 3, and the respective defects of the cold flow properties such as CP, PP, and CFPP on the performance of the engine system. Therefore, CP, PP, and CFPP are the key parameters related to biodiesel crystallization, which depends on the feedstock and types of fatty acids present. For instance, coconut, palm oil, and palm kernel-based biodiesel can be susceptible to high cold flow due to many SFAs. Whereas feedstocks containing a high amount of UFA such as Soybean oil (SBO), cottonseed oil (CSO), and poultry fat (PF) based biodiesel, are more prone to oxidation, which during storage beyond 4 °C formed precipitates [62]. This precipitation of large crystals leads to fuel starvation and operability problems [63], and increases (0.5–1 mm size) linearly as the temperature drops, thereby achieving PP [64]. Progressively, agglomeration of the crystals begins, and the fuel flow systems cease, causing fuel filter lines to clog [65]. According to Magalhães et al. [66], biodiesel's susceptibility to performance problems in cold temperatures is linked to the chemical composition of the fatty acids. Thus, the choice of suitable feedstock is critical to the longevity of biodiesel, especially in a colder environment. Measuring CFPP, CP and PP are considered key parameters for evaluating the crystallization behaviour of different biodiesels. The high melting point at lower temperatures demonstrated by SFAs brings about ease of crystal formation in biodiesel made of such feedstocks [67]. This crystallization occurred at a lower temperature than that of a visible crystal (CP), causing fuel deprivation and consummation problems as freeze substances clog fuel lines and filters.

**Table 3:** A literature survey on the effects of the cold flow behaviour of a different type of biodiesel on engine performance.

S/No.	Biodiesel	Flow property	Various effects when used in engine	References
1.	SBO-BD, RSB, CSB, PB, JME	CP, CFPP	• They formed precipitates during storage at 4 °C, leading to difficulty in engine starting particularly in low ambient regions	[69]
2.	Canola biodiesel	Cold soak filterability	• Biodiesel made from this feedstock resulted in the obstruction of fuel lines and filter	[70]
3.	PEB	CP, PP, CFPP	• Peanut biodiesel quickly forms wax and thickens, which subsequently blocks filters and fuel lines.	[71]
4.	Vegetable oil and animal fat biodiesel.	CFPP	• The high proportion of UFAs present in vegetable and animal oils led to incomplete combustion due to high viscosity and cetane number.	[72]
5.	WCO–B	CFPP	• WCO–B contained predominantly SFA, which in turn causes plugging and gumming of filters owing to poor cold flow.	[73]
6.	TOB	CFPP	• This typically chokes the fuel lines and restricts free flow.	[74]
7.	COB	CFPP	• Easily form a bunch of crystals that affect the ease of engine starting	[75]
8.	Castor biodiesel	CP, PP	• The high possibility of crystal formation at low temperatures leads to flow restriction and fuel starvation.	[76]
9.	Ural crude oil mixed with diesel	CP, CFPP	• Biodiesel made from Ural oil has negligible effects on cold flow properties.	[77]
10.	JME	CP, CFPP	• JME enhanced poor cold flow by overcoming the engine starting problems in a colder environment.	[78]
11.	PM, TAOB, RSB	CP, CFPP, PP	• PB and TAOB performed poorly in cold weather while RSB performs better under cold conditions.	[79]
12.	POB, JME, and Castor biodiesel	CP	• These quickly block fuel supply lines and clog filters, resulting in poor ignition and difficulty in starting.	[80]
13.	Vegetable oil biodiesel	CP	• This formed insoluble and gums that block fuel lines, injectors and also affect spray characteristics.	[81]
14.	*Pongamia* biodiesel	CP, PP	• Solidification occurs which traps oil and restricts free flow, leading to poor atomization.	[82]
15.	SB0–B, PFB, and CSB	CFPP, CP, PP	• Quickly formed a precipitate that blocks fuel lines.	[83]
16.	PEB	CFPP, CP, PP	• Crystallization occurs leading to fuel lines and filter plugging.	[84]
17.	PFB	CFPP, CP	• Solidified and no longer pumpable, leading to fuel lines blockage.	[85]
18.	SBO-B	CFPP, PP, CP	• Low volatility and incomplete combustion due to high viscosity.	[65]
19.	MME	CP, PP	• Solid crystals quickly develop and agglomerate clogging.	[86]
20.	POB	PP, CP	• Formed some impediments to free flow on the engine in cold weather.	[87]

SBO-B = soybean biodiesel; RSB = rapeseed biodiesel; COB = corn oil biodiesel; CSB = cotton seed biodiesel; POB = palm biodiesel; PFB = biodiesel; JME = jatropha methyl ester; MME = mahua methyl ester; PEB = peanut biodiesel, TOB = tabocco oil biodiesel, TAOB = tallow oil biodiesel.

### 1.5 Remediation measures

Issues related to biodiesel's poor oxidation stability and cold flow properties can be addressed through physical, chemical, and genetic moderation. Therefore, the desirability of biodiesel quality such as improved oxidative stability, better cold flow behaviour and considerably reduce emissions, can be achieved inherently by modifying the fatty acid composition through the approaches mentioned above [68]. Nevertheless, these approaches equally have their portion of downsides. For instance, according to Lanjekar & Deshmukh [15], physical methods like winterization and fractionation produce biodiesel with poor oxidative stability, thus non-viable as it later requires improver addition (antioxidants). The chemical method (hydrogenation) is often not feasible in many cases due to the formation of biodiesel with poor cold flow properties, leading to fuel starvation and flow restriction [57]. Reformulation by blending biodiesel with another biodiesel made of different feedstock affects the fuel properties due to variation in fatty acid composition. Whereas blending with mineral diesel is presumed expensive and non-sustainable [66]. Also, the addition of branched-chain fatty alkyl esters significantly affects cold flow properties by forming precipitates and crystals, thereby clogging fuel lines and blockage of filters [67]. Moreover, much work has been done on the use of antioxidants (natural and synthetic) to enhance oxidative stability, but little was done to improve the cold flow behaviour such as CP, PP, and CFPP of biodiesel despite being one of the most effective and efficient methods [5]. Despite that, they have no effects on CP but significantly provoke PP, especially when the crystals adhere to each at a very low temperature.

## 2 Antioxidants

Antioxidants are compounds that inhibit self-oxidation by delaying the generation of oxidants or by breaking the reproduction of free radicals through different processes such as scavenging the species that institute peroxidation, destroying peroxides, chelation by metal ions to delay propagation, terminating singlet oxygen to avert generation of peroxides and cutting off an autoxidation chain reaction [88]. The potency of an antioxidant addition to biodiesel depends on several factors, including the fatty acid profile of FAMEs, the number of natural antioxidants present, solubility, type of chemical structure, activation energy, the position of hydroxyl groups, and redox-reaction potentials which often determines its strengths to resist storage conditions such as light, air, heat, and metal ions [89]. The addition of antioxidants to biodiesel is a suitable way to quickly eliminate self-oxidation owing to their ability to hold back oxidation through several processes. Consequently, the current trend indicates that the use of antioxidants to arrest oxidation stability and maintain a tolerable limit of the fuel quality parameters has become a plausible route to mitigating the effects of biodiesel instability [90].

Natural antioxidants derived from plants are mainly phenolics that retard autooxidation by attacking the reactive oxygen species through various mechanisms such as hydrogen atom transfer (HAT), single electron transfer (SET), and single proton lost electron transfer (SPLET) [91]. Many naturally occurring flavonoids, phenolics, and carotenoids, such as quercetin, gallic acids, tocopherols, ascorbic acid, curcumin, vanillin, lycopene, cinnamic acid, etc, are present in fruit, vegetables, seed, and root among others in several plant species [92]. Despite the promising features of these natural antioxidants, researchers have not done much to explore their potential for enhancing the stability of biodiesel [93].

The use of synthetic antioxidants to arrest oxidation in biodiesel and other fuels has been widely reported [94]. They are phenolic compounds such as BHT, PY and ethoxyquin, BHA, TBHQ, PG, octyl gallate, dodecyl gallate, pyrogallol, and ethoxyquin [52]. These antioxidants have proven to be excellent additives in refined petroleum distillates like gasoline and low-level biodiesel blends but poorly in high-level biodiesel blends. Despite being extensively used to improve the stability of biodiesel, they are more functional in biodiesel made from animal fat than vegetable oil [27]. It is worth noting that poor thermal stability, partial solubility, high toxicity, volatility, and cost are some of the downsides of synthetic antioxidants that saw they are phased out in use [95]. However, it is worth noting that antioxidants' chemical structures determine their stability and the nature of their reaction mechanism [96]. Table 4 presents standards physicochemical properties such as bond dissociation enthalpies (BDE), molecular weight, solubility, and melting points, as factors that determine the structure-activity relationship of antioxidants. Hence, antioxidants with low BDE, higher molecular weight, multi hydroxyl groups (polyphenols), and readily soluble were effective in free scavenging capacity [88].

**Table 4:** Physicochemical properties of some antioxidants (https://pubchem.ncbi.nlm.nih.gov).

S/N	Name	PubChem CID	Molecular formula	Molecular weight (gmol^−1^)	Number of OH group	Solubility in water/alcohol	Melting point (°C)
1.	Curcumin	969516	C_12_H_20_O_6_	368.38	3	SS/CS	30–32
2	Black pepper	638024	C_17_H_19_O_3_	285.34	0	SS/SS	130
3	Cinnamon	637511	C_9_H_8_O	132.16	1	SS/miscible	-7.5
4.	Grape seed extract	78577443	C_32_H_30_O_11_	590.6	9	IS/IS	-
5.	α-tocopherol	14985	C_29_H_50_O_2_	430.7	0	IS/CS	3.0
6.	Eugenol	3314	C_10_H_12_O_2_	164.2	0	SS/SS	-7.5
7.	Rosemary	5281792	C_18_H_16_O_8_	360.31	5	SS/SS	-20
8.	Carvacrol	10364	C_10_H_14_O	150.22	1	IS/SS	1.0
9.	CA	689043	C_9_H_8_O_4_	180.16	4	SS/SS	225
10.	Sesamol	68289	C_7_H_6_O_3_	138.12	1	SS/PS	62–65
11.	FA	445858	C_10_H_10_O_4_	194.18	2	SS/SS	168–172
12.	β-carotene	573	C_40_H_56_	536.9	0	IS/SS	180
13	Ginger extract	6850776	C_35_H_52_O_6_	568.8	1	SS/CS	-
14.	Citric acid	311	C_6_H_8_O_7_	192.12	4	CS/SS	153
15.	Quercetin	5280343	C_15_H_10_O_7_	302.23	5	IS/CS	316.5
16.	TBHQ	16043	C_10_H_14_O_2_	166.22	2	IS/CS	128
17.	BHA	31404	C_15_H_24_O_2_	220.35	1	IS/SS	71
18.	BHT	24667	C_22_H_32_O_4_	360.5	1	IS/SS	70
19.	PY	1057	C_6_H_3_O_3_	126.11	3	SS/CS	133
20.	PG	4947	C_10_H_12_O_5_	212.2	3	SS/CS	130

SS = slightly soluble, CS = completely soluble, IS = insoluble soluble.

### 2.1 Effects of antioxidants on biodiesel oxidative stability

The use of antioxidants to counter the effects of oxidation stability has been widely explored despite the shadow understanding of how the chemistry of antioxidants impacts fuel stability. Considerably, a satisfactory level of understanding now exists as documented by many pieces of literature [88]. Despite many steps placed forward nowadays by researchers, there is still a need to dwell much on exploiting the potential of vast natural antioxidants to retard oxidation in biodiesel. This is an attempt to avoid the use of expensive, toxic, and insoluble synthetic antioxidants, while at the same time harnessing the potential of vast unexploited natural product compounds. However, the effectiveness of antioxidants varied depending on their type, the concentration and or the ratio applied, the feedstock from which the biodiesel is produced, and the proportion of the blending mixture. Table 5 summarizes the measured induction period of raw and antioxidant blended biodiesel made from different feedstocks.

**Table 5:** Summary of literature survey on the oxidation stability (IP) of raw and antioxidants blended biodiesel.

S/N	Biodiesel	Antioxidant's Blend(s)	Antioxidant(s) Concentration (ppm)	Induction Period (h)	References
1	WCO–B		-	2.0	[36]
2		WCO–B + turmeric	2000	18.1	[36]
3		WCO–B + black pepper	✓	11.0	[36]
4		WCO–B + cinnamon	✓	9.0	[36]
5		WCO–B + watermelon seed	✓	5.0	[36]
6	WCO–B		-	3.0	[98]
7		WCO–B + Grape seed oil	5000	3.61	[98]
8		WCO–B + Vitamin E	✓	3.42	[98]
9		WCO–B + Eugenol	✓	4.90	[98]
10	B10		-	6.5	[99]
11		B10 + Rosemary	1000	9.4	[99]
12		B10 + Oregano	✓	8.2	[99]
13		B10 + Basil	✓	8.02	[99]
14		B10 + Rosemary + oregano	✓	10.18	[99]
15		B10 + Rosemary + basil	✓	9.43	[99]
16		B10 + Oregano + basil	✓	9.2	[99]
17	SBD		-	4.97	[100]
18		SBD + Curcumin	NI	8.03	[100]
19		SBD + β-Carotene	NI	4.50	[100]
20		SBD + Curcumin + β-Carotene	NI	7.19	[100]
21	SBD		-	4.0	[105]
22		SBD + CA	2000	14.4	[105]
23		SBD +SE	✓	9.0	[105]
24		SBD + WTP	✓	15.7	[105]
25		SBD + LTP	✓	9.0	[105]
26		SBD + FA	✓	4.0	[105]
27		SBD + BHA	✓	7.0	[105]
28	Croton biodiesel		-	4.0	[102]
29		Croton biodiesel + BHA	1000	6.8	[102]
30		Croton biodiesel + PG	✓	8.2	[102]
31		Croton biodiesel + PY	✓	10	[102]
32	SBD		-	0.7	[23]
33		SBD + BHA	>8000	>3.0	[23]
34		SBD + PG	>6000	>3.0	[23]
35		SBD + TBHQ	3000	>6.0	[23]
36		SBD + PY	1500	<3.0	[23]
37		SBD + BHT	✓	<3.0	[23]
38		SBD + α-tocopherol	✓	<3.0	[23]
39	UFO–B		-	6.0	[106]
40		UFO–B + PG	250	>6.0	[106]
41		UFO–B + BHA	500	>6.0	[106]
42		UFO–B + vitamin E + BHT + TBHQ	1000	>6.0	[106]
43	RSB		-	<6.0	[107]
44		RSB + BHA + BHT + citric acid	400	>6.0	[107]
45	*Karanja biodiesel*	PY	100	16	[108]
46	*Pongamia* biodiesel	PY	3000	34	[109]
47	MOME	PY + PG	100	6.23	[110]
48	JME	TBHQ + BHA	600	>6.0	[111]
49	JME	PY	250	>6.0	[112]
50	JME	PY	100	>6.0	[113]

B10 = 10% blended biodiesel; JME = Jatropha Methyl Ester; SBD = Soybean biodiesel; UFO–B = Used frying oil biodiesel.

MOME = *Moringa* oil methyl ester; RSD = Rapeseed biodiesel; WCO = Waste cooking oil.

Nagarajan & Narayanasamy [36], synthesized biodiesel using waste cooking oil (WCO) and blended it with turmeric, black pepper, cinnamon, and watermelon seed extracts respectively, using a concentration of 2000 ppm. The stability index of the raw and the respective blended biodiesel with the extracts was determined using the Rancimat method at 110 °C. As presented in Table 5, the WCO-biodiesel blended with turmeric improves the stability most by raising the IP to 18.1 h from the initial 2.0 h displayed by the raw WCO-biodiesel. This may be attributed to the fact that curcumin is the main constituent of turmeric [97]. Similarly, black pepper and cinnamon extracts also improved the stability of the biodiesel by increasing the IP to 11.0 and 9.0 h, respectively. Both IPs recorded were above the minimum 6.0 h stipulated by European standard EN 14112. However, only the sample doped with watermelon demonstrated inferiority below the minimum standard (5.0 h). Therefore, their antioxidants strengths at 2000 ppm follow the order: turmeric > black pepper > cinnamon > watermelon seed. Similarly, Ramos et al. [98], examined the effectiveness of vitamin E, grape seed oil, and eugenol in improving the IP of biodiesel derived from WCO. According to the authors' findings, all the extracts exhibited poor antioxidative effects by not attaining the minimum biodiesel IP standard (EN 14112) of 6.0 h. This corroborates with the observation made by Varatharajan & Pushparani [27], that most individual natural antioxidants demonstrate poor performances even when they are applied at higher concentrations. Research conducted by Spacino et al. [99], stabilizes biodiesel (B10) using antioxidants extracts of rosemary, basil, and oregano in mono and binary blends at 1000 ppm. According to the authors, all the extracts showed enhanced oxidation resistance of the biodiesel. Nevertheless, the binary blends of the antioxidants demonstrated improved oxidation stability of the biodiesel than the mono blend as shown in Table 5.

Similarly, Sousa et al. [100], examined the oxidation stability of raw and antioxidant blended soybean biodiesel (SBD), with curcumin and β-carotene at unspecified concentrations. The reaction was monitored as a function of storage time for six (6) months at 110 °C and the IP was measured using Rancimant and Petro-OXY methods. The results show that Raw SBD indicated an IP of 4.97 h, below the ASTM standard specification (6.0 h), thus the need for antioxidant additions. However, SBD blended with curcumin, β-carotene, and a mixture of both, gave IPs of 8.03, 4.50, and 7.19 h, respectively. According to the authors, curcumin acted as a powerful antioxidant by enhancing the stability of biodiesel by up to 83 %.

On the contrary, β-carotene acts as a pro-oxidant, demonstrating poor stability. This may be attributed to the lack of hydroxyl groups in β-carotene, affecting its reactivity and antioxidant capacity [101]. In another research, Osawa et al. [102], studied the effect of antioxidants oxidative stability on improving croton biodiesel and its blends with petro-diesel using the Rancimant method. However, after adding the antioxidants at concentrations of 1000 ppm, the biodiesel IP increased to 6.8, 8.2, and 10 h with blends of BHA, PG, and PY, respectively. A similar trend was reported by Yang et al. [23], using the same type of antioxidants with PY addition. This can be upheld since, not in all cases, the relation between the increase in concentrations and antioxidant activity is linear, particularly for phenolic antioxidants. Generally, antioxidants’ chemical structure, stability, solubility, blending mode, and dosage ratio determines their ability to scavenge free radical. However, Tan et al. [103], revealed that biodiesel made from highly UFAs feedstock is more susceptible to poor oxidative stability. This is because hydrogen atoms belonging to bis-allylic carbons are more loosely held than those in allylic carbon and thus can be lost easily to initiate oxidation. Hence, this may be responsible for the oxidization as demonstrated by Karanja and Moringa oils biodiesel than Jatropha methyl esters. It can be summed up that natural antioxidants such as turmeric, WTP, CA, black pepper, cinnamon, and rosemary extracts demonstrated excellent antioxidants activity, by delaying the IP from (6.0–18 h). While grapeseed oil, watermelon seed, α-tocopherol, and eugenol extracts displayed poor activity (3.0–5.0 h), below 6.0 h ASTM standard for IP of biodiesel, even at higher concentrations [104].

### 2.2 Effects of antioxidants addition on biodiesel cold flow properties

The improvement of biodiesel cold flow using antioxidant additives is regarded as a conventional method that is sustainable and cost-effective. These additives improve the cold flow by co-crystallizing the fuel crystals and decelerating further crystal growth. Typically, this involves restraining the wax crystals from growing bigger and preventing further crystal enlargement at low temperatures [114]. According to Muniz et al. [53], polymeric and phenolic compounds are excellent PP depressant and antioxidant additives respectively. Therefore, the chemical structures of these additives consist of a hydrocarbon series that is liable to co-precipitate with the hydrocarbon chain of the fuel and prevent the development and solidification of the wax crystals [115]. During nucleation disruption of the crystal structure occurred and formed from a three-dimensional shape that is narrow and long-pointed, thereby preventing fuel filters from blockage and retarding the crystal growth to avoid solidifying. The CP, PP, and CFPP of coconut biodiesel (COB) were very high and liable to crystals formation due to too much quantity of SFAs. This became evident by meeting the 6.0 h IP of EN 21214, signifying better oxidative stability but poor cold flow. The addition of ginger and pepper raises the IP by just 3% and is steadily higher using garlic. Hence, 3% was the maximum thermal stability the extracts can achieve [17].

Similarly, adding 0.5 wt % to 1.0 wt % of EVAC and 0.04 % PMA improver typically reduces the CFPP by 2 °C, but a much lower decrease in CP and PP of WCO-biodiesel (Table 6), as reported by Wang et al., [116]. The use of the distillation method to purify biodiesel significantly reduces the number of antioxidants present, affecting the protection shield [117]. This further enhances the poor cold flow by lowering the number of SFAs, leading to improved CP, CFPP, and PP of the biodiesel [118]. Studies conducted by Senthil et al. [119], examined the IP, CP, and PP of jasmine methyl ester (JSME 100) and JSME 20 blends. The resulting improvement in IP and decrease in CP and PP of JSME 20 compared to the unblended JSME100 could be attributed to the low UFA profile in the mineral diesel, which increases the stability and flow properties of the diesel-Jamun biodiesel blend. Kleinberg et al. [120], reported using cashew nutshell liquid to improve the IP and CP of beef tallow biodiesel (BTB). The polymerization and poor solubility of CNSL in water (1.1 mg/L at 20 °C) were among the factors mainly responsible for its enhanced antioxidative effect [121].

**Table 6:** Impacts of antioxidants blended and raw biodiesel on cold flow and oxidative stability (IP) according to some pieces of literature.

S/N	Raw and antioxidant's blended biodiesel	Viscosity at 40 °C (mm^2^/s)	Parameters	CP (°C)	PP (°C)	IP (h)	References
			Density (kg/m^3^)				
1.	Raw COB	20.03	861	14	23	6.46	[17]
2.	COB + ginger extract	18.51	-	12	11	7.5	[17]
3.	COB + pepper extract	18.82	-	12	13	7.2	[17]
4.	COB + garlic extract	18.51	-	10	8.5	8.0	[17]
5.	COB *+ T. cordifolia* extract (1000ppm)	7.0	841	7	6	3.5	[122]
6.	CPOME	4.50	875	14.5	15	25.7	[117]
7.	DPOME	4.42	878	13.6	15	3.52	[117]
8.	α-tocopherol + CPOME	-	-	14	11	25.7	[117]
9.	α-tocopherol + DPOME	-	-	10	9.0	6.17	[117]
10.	WCO–B	4.8	-	-8	-11	3.8	[116]
11.	WCO–B + 0.04 % PMA	4.86	-	-9	-19	6.7	[116]
12.	WCO–B + 0.04 % EVAC	4.92	-	-8	-17	6.6	[116]
13.	WCO–B + 0.04 % PAO	4.73	-	-9	-14	6.5	[116]
14.	WCO–B + 0.04 % PAH	4.76	-	-8	-12	2.88	[116]
15.	WCO + PMA extract	4.59	860	14	13	-	[87]
16.	JSME100	4.72	872	19	11	3.0	[119]
17.	JSME20	4.5	855	15	8.0	8.0	[119]
19.	JSME + *Albizia Lebbeck (*1000 ppm)	4.0	835	11.5	9	18.5	[119]
20.	JSME + *Melia Azedarach* (1000 ppm)	4.2	840	12	8	15.2	[119]
21.	JSME + *Psidium Guajava* (1000 ppm)	4.3	842	12	7	14.6	[119]
22.	Canola biodiesel B100	2.68	855	-4	-5	8.8	[9]
23.	Raw canola BD	-	-	-12	-16	-	[123]
24.	Canola BD + 1% poly (Lauric methacrylate)	-	-	-12	-46	-	[123]
25.	Raw palm oil	39.4	919	25.2	23.6	-	[87]
26.	Palm-Biodiesel	4.43	880	21	19.7	-	[122]
27.	BTB	4.8	871	12	-	0.55	[120]
28.	BTB + Natural CNSL	5.5	874	9	-	4.9	[120]
29.	*Pongami*a *pinnata* biodiesel	5.76	890	20	19	1.8	[124]
30.	Conventional diesel	1.3–41	700–790	5–15	15–35	45	ASTM D975

BD: biodiesel; WCO–B: waste cooking oil biodiesel, COB: coconut biodiesel, CPOME: crude palm oil methyl ester.

PMA: polymethyl acrylate, EVAC: ethylene-vinyl acetate copolymer, PAH: polymaleic anhydride.

### 2.3 The mechanism for the chemistry of antioxidants addition

The process of antioxidants inhibition mechanism can be filed as free radical terminators, metal ion chelators, and oxygen scavengers, all of which are tailored toward improving biodiesel resistance to oxidation by eliminating free radical and decelerating propagation chain. Thus, initiation, propagation, and termination represent the three phases of autooxidation.

Antioxidants' inhibitory role in the initiation stage

At the initiation stage, the loss of hydrogen atom from either allylic or bisallylic carbon of the UFA chain, to produce an intermediate called carbon-centred free radical (R^•^) as shown in Eq. (12), is instigated by the initiators or oxidation actors such as heat, light, metal ions, humidity or atmospheric oxygen.

Eq. 12 RH + I → R^•^ + IH

The above reaction can be quenched by adding primary or phenolic antioxidants (AOH), called free radical terminators, and transforming them into thermodynamically fixed products. Comparatively, the secondary AOH or the metal chelators (antioxidant synergists) often decelerate the rate of oxidation reaction through complex formation with the metal ions, preventing catalytic reaction and subsequent biodiesel degradation [125].

Antioxidants' inhibitory role in the propagation stage.

The produced carbon-based free radical (R^•^) at the initiation stage, is greatly reactive and can quickly react with accessible oxygen to form peroxyl radicals (ROO^•^) (Eq. 13).

Eq. 13 R^•^ + O_2_ → ROO^•^

The peroxyl radical (ROO^•^) is less reactive than the earlier produced radical (R^•^), but it is sufficient to abstract another hydrogen having a low bond dissociation enthalpy to produce hydroperoxide (ROOH^•^) and an extra free radical (R^•^) as shown in Eq. (14).

Eq. 14 ROO^•^ + RH → ROOH + R^•^

Therefore, the chain keeps propagating continuously, but the presence of phenolic or chain-breaking antioxidants (AOH), cut off the autoxidation chain reaction by deactivating the ROO^•^ and terminating the carbon-centered free radical chain (R^•^), thereby producing an antioxidant radical (AO^•^) [126]. This antioxidant radical is stabilized and inert, which cannot initiate or propagate the oxidation process. Hence, the chain reaction is ceased and is subsequently consumed as shown in Eqs. (15), (16), (17), and (18).

Eq. 15 ROO^•^ + AOH → ROOH + AO^•^

Eq. 16 R^•^ + AOH → RH + AO^•^

Eq. 17 ROO^•^ + AO^•^ → ROOA

Eq. 18 R^•^ + AO^•^ → ROA

The above reactions release heat to the surrounding and as the bond dissociation energy increases, the activation energy and efficiency of the antioxidants increases. Consequently, the antioxidative properties of phenolic and non-phenolic compounds usually depend on the position of their functional groups, which serve a vital role in preoccupying and neutralizing free radicals, quenching singlet and triplet oxygen, and or breaking down peroxide. Thus, phenolic antioxidants are excellent antioxidative candidates, owing to their low activation and bond dissociation energies, allowing them to easily donate hydrogen atoms and form radical intermediates that are stabilized by resonance. While for the non-phenolic compounds, the presence of continuous overlap (extended conjugation) avails the compound with an opportunity to transfer electrons quickly and becomes stabilized by resonance. Moreover, the substituents at the ortho, para, and meta position increase their electron density by inductive effect and strengthen the steric hindrance around the free radicals. This reduces the rate of possible propagation and ultimately increases the stability of the radicals through intermolecular hydrogen bonding.

## 3 Research trends and sustainability of using antioxidants for biodiesel stabilization

The application of antioxidants to increase biodiesel resistance to oxidation stability and enhance biodiesel cold flow dates back decades ago. With this, many researchers [127, 128] reported using antioxidants as a cost-effective and sustainable route for improving biodiesel performance. However, according to a literature overview search for ‘‘antioxidants’’, ‘‘oxidation stability’’, ‘‘cold flow properties’’ and ‘‘biodiesel’’, Knothe & Dunn [129], was found to be the most versatile researcher in this wing, with 826 citations, followed by Dunn [130] (498 citations), and Pullen & Saeed [44], having 440 citations. At the same time, several other authors were cited below 400. However, a greater percentage of the antioxidants were utilized to protect edible oil against oxidation reactions in the food industry as evidenced by the literature findings, where over 2419 publications were reported between 1956 to 2022. Accordingly, Choe & Min [131], published a comprehensive review in food science and food safety, communicating the mechanisms and factors behind edible oil oxidation, and had the highest citations (1043) [3].

Consequently, the most widely reported and used antioxidants were synthetic, with PY being the most effective and frequently utilized for biodiesel oxidative stability, followed by PG, TBHQ, BHA, BHT, and many others in a few reported cases [132]. Doubtlessly, issues linked to human safety associated with using synthetic antioxidants, particularly their high toxicity and volatility, are some of the fragilities that accounted for their current phase-out, especially in the food industry [133]. Moreover, many of these antioxidants were reported to be only active at higher concentrations of 1000 ppm and above. Hence, considering the cost of these antioxidants coupled with their partial solubilities resulted in engine deposits, which subsequently clogged filters, blocked fuel lines, and ultimately heightened maintenance costs. Therefore, it will be inferred that the use of this class of antioxidants is not sustainable and economically viable [134]. This prompted the consideration of natural antioxidants as better candidates for biodiesel stabilization.

On the other hand, many natural antioxidants, notably polyphenols, flavonoids, carotenoids, and amines, were reported as additives for biodiesel shelf life improvement due to their availability of hydrogen atoms for free radical scavenging, renewability, solubility, and low cost [135]. Unfortunately, most of these natural antioxidants were found to be ineffective in improving the biodiesel IP, especially when used in mono blends [136]. It is apparent from the pieces of literature X-rayed, that researchers have not done much towards exploring the potency of this class of antioxidants for cold flow improvement of biodiesel. Another steam debate is the fact that excessive exploitation of consumable natural products (edible) for use as antioxidants additives, poses a greater threat to food security by aggravating the food versus fuel clash. Hence, an attempt to make the process resource competition-free, labour-saving, and economically viable accounted for the exploration of diverse research-based, and profitable strategies. This is in line with industries' targets of ensuring sustainable production at the expense of readily available and low-cost raw materials. In this context, few studies [6], reported the use of non-edible sources of antioxidants for oxidative stability and poor cold flow improvement. Therefore, it is worth noting that from the outlined investigation discovered in the literature, the use of synthetic antioxidants and edible types of natural antioxidants, dominated the research sphere. Thus, the need for the transitioning to the redundant non-edible sources of natural antioxidants especially biowaste, to ensure sustainability and economic viability of using antioxidants for biodiesel's oxidative and poor flow enhancement.

## 4 Limitations and future recommendations

Augmenting petroleum-derived fuels with renewable fuels has received all-inclusive attention in recent years by swinging the pendulum of implementing several energy policies, particularly with energy from biomass as one of the most oscillating options. It is no doubt that non-sustainability, high toxicity, and bulk of GHG emissions diminish the lustre of fossil-based fuels. Therefore, biodiesel as an alternative to mineral diesel remained scalable and promising, but poor oxidation stability and cold flow are still the gridlocks that negate its broader acceptance. From the reviewed pieces of literature, oxidation stability, poor cold flow, and NOx emissions put forward opposing conditions on the fatty acid composition of biodiesel made from both edible and non-edible oil, thus the need to dwell much on exploring micro algae-based oil and waste from sludge as feedstock for biodiesel. This saves cost, especially from using waste as feedstock, and deviates from the inconsistencies in the fuel quality parameters, occasioned by a variation in fatty acid composition from the most edible and non-edible feedstocks. However, antioxidants have been identified as a cost-effective and plausible option to counteract the effects of biodiesel's oxidation stability and poor cold flow. The following factors should be considered in selecting an excellent antioxidant for effective performance.

• Antioxidants with good solubility, non-toxic, and effective at low concentrations
• Antioxidants with a low bond dissociation energy (<40 KJmol^-1^) are more suitable since they can quickly transfer hydrogen atoms to the free radical.
• Higher molecular weight antioxidants (polyphenols) are favoured for the everlasting storage of biodiesel owing to enough hydrogens for donation. Whereas low molecular weight antioxidants have ease of migration freely to reach the initiation site, these types of antioxidants are highly volatile and thus evaporate quickly.
• Antioxidants with bulkier polyhydroxy groups performed better than mono phenolic-based. However, the activity does not improve beyond the three hydroxy groups.
• Antioxidants with a longer aliphatic chain (carotenoids) or those with a greater number of benzene rings have good heat resistance.

However, having identified antioxidants as potent improvers of biodiesel oxidative stability and better flow enhancers as documented by many reviews, that is not sufficient to scale up its use as a suitable additive for biodiesel stabilization, as other legitimate pressing issues remained unchecked. For instance, the economic viability and sustainability of synthetic antioxidants are still debated, thus the need to redirect towards the exploration of bio-based materials as antioxidants sources. In this context, decomposable bio-wastes from restaurants, residentials, and vegetable markets, can be attractive sources of antioxidants. Interestingly, converting these antioxidant sources to an antioxidative agent would be sustainable, economically viable, and environmentally friendly, tailored to convert wastes to value-added products in line with the circular economy. Moreover, this will step up waste management, safeguard the environment by minimizing pollution and other emissions like nitrous oxide and GHG, and at the same time hold back resource competition for using edible substances.

Therefore, to unlock the potentiality of using antioxidants as a promising route for modifying the fatty acid composition and a promoter of biodiesel stabilization, this review suggests that future work should be tailored towards exploring reusable biowastes as antioxidative agents for improving biodiesel cold flow and oxidative stability. Other important aspects that need to be researched include a thorough investigation and in-depth analysis of the effects of antioxidants concentration on emission discharged from engines, rate of antioxidant blended biodiesel's evaporation in a heated engine, influence of antioxidants on reduced smoke and HC emissions, and influence of antioxidants on reduced biodiesel consumption and the activation temperature. Currently, no work appears on the areas mentioned above, hence the need for future researchers to dwell on these underlying aspects.

## 5 Conclusions

The balanced growth in the world population day by day, coupled with increasing emissions from fossil fuel combustion, propelled the quest for clean and sustainable energy to quench the growing energy thirst. As such, biodiesel is placed forward as clean, renewable energy that is used without modification, and this pressured its interest globally especially looking at its non-toxicity, biodegradability, renewability, and environmentally benign nature. Nevertheless, the oxidization vulnerability of biodiesel and poor cold flow was mainly plagued by UFAs and SFAs present in the feedstocks. While other oxidation actors such as storage temperature, metal containers, air, and sunlight exposure also initiate and propagate oxidation stability in biodiesel but to a lesser extent. For instance, when biodiesel oxidizes, products such as aldehydes, ketones, and smaller chain acids are formed, which later undergo several reactions such as polymerization and aldol condensation to generate insoluble gums and sediments. These substances can increase viscosity, density, and peroxide value, which ultimately leads to poor atomization and low-quality ignition. Although the oxidation scenario cannot be prevented entirely but can be substantially slowed down using antioxidants to improve the biodiesel's oxidative stability. Hence, increasing resistance to oxidative degradation during storage and processing discussed in this paper is an increasingly vital topic for promoting the longevity and feasibility of biodiesel.

Conversely, poor cold flow is another major issue of biodiesel, compromising the ease of engine starting particularly in low-temperature environments or colder regions. Against this backdrop, many researchers established that the poor cold flow of biodiesel is the leading cause of the plugging of fuel lines and filters, leading to fuel starvation and poor ignition. As a result of this problem, incomplete fuel combustion is experienced, leading to difficulty in engine starting. To get rid of these problems, researchers recommend using suitable feedstock for biodiesel production, and the focus shifted from edible and non-edible feedstocks to microalgae and sludge wastes addressed as third and fourth-generation feedstocks, respectively. This reduces the number of fatty acids predominantly found in edible and non-edible feedstocks. Contrarily, such feedstocks require a high purity process, making it tedious and time-consuming, costly, and unpleasant smelt and the high possibility of microbial degradation are some of the downsides threatening its sustainability. Other proactive steps include using opaque storage containers to avoid light exposure, blending it with petroleum diesel, and adding antioxidants, among other approaches. Most importantly, establishing a clearer understanding of biodiesel's oxidative stability and cold flow on engine performance is an area of research that needs to be expanded at the current rate.

## Declarations

### Author contribution statement

All authors listed have significantly contributed to the development and the writing of this article.

### Funding statement

This work was supported by HICOE Centre for Biofuel and Biochemical Research (015MA0052) and Yayasan Universiti Teknologi PETRONAS (015LC0-042, 015LC0-438).

### Data availability statement

Data included in article/supplementary material/referenced in article.

### Declaration of interests statement

The authors declare no conflict of interest

### Additional information

No additional information is available for this paper.
